# COH-SR4 Reduces Body Weight, Improves Glycemic Control and Prevents Hepatic Steatosis in High Fat Diet-Induced Obese Mice

**DOI:** 10.1371/journal.pone.0083801

**Published:** 2013-12-20

**Authors:** James Lester Figarola, Preeti Singhal, Samuel Rahbar, Bogdan Gabriel Gugiu, Sanjay Awasthi, Sharad S. Singhal

**Affiliations:** 1 Departments of Diabetes and Metabolic Diseases Research, Beckman Research Institute, City of Hope National Medical Center, Duarte, California, United States of America; 2 Immunology and Mass Spectrometry and Proteomics Core, Beckman Research Institute, City of Hope National Medical Center, Duarte, California, United States of America,; 3 Medical Oncology, Beckman Research Institute, City of Hope National Medical Center, Duarte, California, United States of America; Roswell Park Cancer Institute, United States of America

## Abstract

Obesity is a chronic metabolic disorder caused by imbalance between energy intake and expenditure, and is one of the principal causative factors in the development of metabolic syndrome, diabetes and cancer. COH-SR4 (“SR4”) is a novel investigational compound that has anti-cancer and anti-adipogenic properties. In this study, the effects of SR4 on metabolic alterations in high fat diet (HFD)-induced obese C57BL/J6 mice were investigated. Oral feeding of SR4 (5 mg/kg body weight.) in HFD mice for 6 weeks significantly reduced body weight, prevented hyperlipidemia and improved glycemic control without affecting food intake. These changes were associated with marked decreases in epididymal fat mass, adipocyte hypertrophy, increased plasma adiponectin and reduced leptin levels. SR4 treatment also decreased liver triglycerides, prevented hepatic steatosis, and normalized liver enzymes. Western blots demonstrated increased AMPK activation in liver and adipose tissues of SR4-treated HFD obese mice, while gene analyses by real time PCR showed COH-SR4 significantly suppressed the mRNA expression of lipogenic genes such as sterol regulatory element binding protein-1c (*Srebf1*), acetyl-Coenzyme A carboxylase (*Acaca*), peroxisome proliferator-activated receptor gamma (*Pparg*), fatty acid synthase (*Fasn*), stearoyl-Coenzyme A desaturase 1 (*Scd1*), carnitine palmitoyltransferase 1a (*Cpt1a*) and 3-hydroxy-3-methyl-glutaryl-CoA reductase (*Hmgcr*), as well as gluconeogenic genes phosphoenolpyruvate carboxykinase 1 (*Pck1*) and glucose-6-phosphatase (*G6pc*) in the liver of obese mice. *In vitro*, SR4 activates AMPK independent of upstream kinases liver kinase B1 (LKB1) and Ca2+/calmodulin-dependent protein kinase kinase β (CaMKKβ). Together, these data suggest that SR4, a novel AMPK activator, may be a promising therapeutic compound for treatment of obesity, fatty liver disease, and related metabolic disorders.

## Introduction

Obesity is a common metabolic disorder that is rapidly becoming a global public health in adult and pediatric populations, affecting more than 500 million people worldwide in 2013 [1]. It is associated with an increased risk of several life-threatening diseases such as type 2 diabetes (T2D), cardiovascular diseases, renal diseases, and multiple types of cancer and may represent a leading preventable cause of death [2], [3]. Obesity is characterized by pathologic growth of adipose tissues to accommodate excess energy intake through an increase in the number (hyperplasia) and size (hypertrophy) of adipocytes [4], and dysregulation of chronic inflammatory and hormonal signaling pathway responsible for regulation of long-term nutrient metabolism, energy balance, appetite and satiety [5], [6].

Maintaining energy balance depends on the efficiency of tightly regulated mechanisms of energy intake and expenditure. Besides the well-established role of nutrients, hormones and hypothalamic neural circuits, evidence has demonstrated that basic cellular metabolic pathways have a major role in the regulation of whole body energy homeostasis. Among them is 5′ adenosine monophosphate-activated protein kinase (AMPK), an energy-sensing enzyme that integrates nutrients, hormones, and stress signals to maintain whole body energy homeostasis [7], [8]. AMPK regulates several proteins involved in glucose and lipid metabolism, including phosphorylation and inactivation of enzymes of fatty acid and cholesterol synthesis, as well as activating ATP-generating processes, including the uptake and oxidation of glucose and fatty acids [9]–[11]. AMPK activation also results in long-term regulation of glycolytic and lipogenic gene expression *via* phosphorylation of transcription factors, co-activators, and co-repressors [7], [12], [13]. Interestingly, knockdown of AMPKα1 or AMPKα2 subunits led to the development of obesity and insulin resistance in mice [14], [15]. Due to its involvement in the regulation of a variety of metabolic processes and its central role in glucose and lipid homeostasis, AMPK has become an attractive drug target for the treatment of obesity, T2D, fatty liver disease, cancer, and other metabolic diseases [11], [16]–[18].

Recent studies have demonstrated that anti-diabetic drugs such as metformin and thiazolidinediones, as well as phytochemicals/nutraceuticals like resveratrol, curcumin, glabridin and berberine act as AMPK activators and exhibit beneficial effects on metabolic disorders including obesity, diabetes, hyperlipidemia and insulin resistance [7], [11], [18]–[24]. Our laboratory recently found some novel small molecules with potential anti-cancer activities while screening for compounds with anti-glycation and anti-inflammatory effects. One of these is COH-SR4 (SR4), which showed strong anti-proliferative effects against a wide variety of human and animal cancers *in vitro* and *in vivo* [25]–[27]. SR4 was recently shown to activate AMPK and also prevents adipocyte differentiation of mouse 3T3-L1 cells [27]. We therefore tested the hypothesis that long-term (6-week) treatment of SR4 in high-fat diet (HFD)-induced obese mouse, would improve the metabolic alterations in this animal model of human obesity, including hyperglycemia, hyperlipidemia and insulin resistance. Additionally, we examined the effects of SR4 on hepatic and adipose AMPK activation, adipose hypertrophy, liver steatosis, as well as analyzed the effects of the compound on various genes associated with hepatic lipid and glucose metabolism. Results of present studies demonstrated that SR4 administration exerted beneficial effects on both glucose and lipid metabolism, which were more likely due to the ability of SR4 to activate AMPK in HFD obese mice. Our data thus suggest that SR4 may be a promising therapeutic compound for treatment of obesity, fatty liver disease, and related metabolic disorders.

## Materials and Methods

### Chemicals and Reagents

SR4 was synthesized according to a previously validated protocol by Dr. Christopher Lincoln at the Chemical GMP Synthesis Facility, Translational Medicinal Chemistry Laboratory, Beckman Research Institute of the City of Hope [25]. Antibodies against AMPKα, pAMPKα (Thr^172^), ACC, pACC (Ser^79^), LKB1 and β-actin were obtained from Cell Signaling Technology (Danvers, MA, USA). STO-609, 5-aminoimidazole-4-carboxamide ribonucleotide (AICAR), and Compound C were from Tocris Bioscience (Bristol, UK). Unless otherwise noted, all other chemicals and reagents were purchased from Sigma-Aldrich (St. Louis, MO, USA).

### Animals, Diets and Study Design

All animal experiments were carried out in accordance with a protocol approved by the Institutional Animal Care and Use Committee (IACUC) of the City of Hope National Medical Center. Nine-week old male C57BL/6J mice were purchased from Jackson Laboratory (Bar Harbor, ME, USA). Animals were housed under standard 12∶12 h light: dark cycle. Animals were fed with either a high fat diet (HFD) (D129492: 60% fat, 20% protein and 20% carbohydrate; 5.24 kcal/g) or a low-fat diet (LFD) (D12450B; 10% fat, 20% protein; 70% carbohydrate; 3.85 kcal/g) from Research Diets (New Brunswick, NJ, USA). Experimental animals were maintained on their respective diets and divided into 3 groups (n = 12 per group): LFD, HFD + vehicle (corn oil), and HFD + SR4. Corn oil and SR4 (5 mg/kg/body weight) were administered orally by gavage at 200 ul total volume three times a week (Mon, Wed, Fri) during the early morning hours for 6 weeks. Body weights and food consumptions were monitored throughout the study. At the end of the study, mice were sacrificed, blood was collected *via* cardiac puncture, and plasma separated subsequently for further analyses. Tissue samples were collected and either snap-frozen and stored at -70°C, or placed in 10% neutral buffer formalin (NBF) for further biochemical and immuno-histochemical analyses, respectively.

### Glucose Tolerance Tests

At the 5-week treatment period, mice were fasted overnight for 16 h. Animals were then given 2 g/kg body weight of D-glucose solution intraperitoneally and tail vein blood was collected serially at 0, 30, 60, 90, and 120 min following challenge. Blood glucose concentration was measured with a glucometer (Accu-Chek Compact Plus, Roche Diagnostics, IN, USA).

### Metabolic Measurements

Plasma insulin levels were determined by Ultra Sensitive Mouse Insulin ELISA kit (Crystal Chem Inc., Downers Grove, IL, USA). Plasma leptin and adiponectin levels were determined by ELISA kits (Life Technologies, Carlsbad, CA). Plasma alanine aminotransferase (ALT) and aspartate aminotransferase (AST) concentrations were quantified using the Vitros 250 Chemistry System (Johnson & Johnson, Rochester, NY, USA) at the Pathology Core Lab, City of Hope, Duarte, CA. Total plasma triglycerides, plasma cholesterol, and hepatic triglyceride levels were measured enzymatically using the Total Triglyceride Quantification and Total Cholesterol Quantification Kits (Cell BioLab Inc., San Diego, CA, USA) according to the manufacturer's instructions.

### Cell Culture

HepG2 (human liver hepatocellular carcinoma) and A549 (human lung adenocarcinoma) cell lines were obtained from ATCC (Manassas, VA, USA), and cultured on EMEM or F-12K medium, respectively, supplemented with 10% fetal bovine serum (FBS), 100 units/ml penicillin, and 100 mg/ml streptomycin. Cells were maintained at 37°C in a humidified atmosphere containing 5% CO_2_. For experimental treatments, cells were seeded into 6-well culture plates overnight and treated with the test compounds at the indicated time periods.

### Quantitative Real-time PCR

Total RNA was isolated from liver samples using the RNEasy kit (Qiagen, Valencia, CA). First strand cDNA was prepared using the High Capacity cDNA Reverse Transcription Kit (Life Technologies). Real-time qPCR was performed on an ABI-7500 fast real time PCR system (Life Technologies) using Power SYBR Green master mix. The list of primer pairs used and their sequences are provided in Table 1. After initial incubation for 2 min at 50°C, the cDNA was denatured at 95°C for 10 min followed by 40 cycles of PCR (95°C for 15 s, 60°C for 60 s). The relative mRNA levels of all genes were quantified using the comparative Ct method [28] with β-actin as an internal control.

**Table 1:** Sequences of primers used in real-time PCR.

Gene	Forward primer	Reverse primer
*Actb*	ACCTTCTACAATGAGCTGCG	CTGGATGGCTACGTACATGG
*Acaca*	CCTCCGTCAGCTCAGATACA	TTTACTAGGTGCAAGCCAGACA
*Cpt1a*	GCTGGAGGTGGCTTTGGT	GCTTGGCGGATGTGGTTC
*Fasn*	GCGATGAAGAGCATGGTTTAG	GGCTCAAGGGTTCCATGTT
*G6pc*	GAGTCTTGTCAGGCATTGCT	GGTACATGCTGGAGTTGAGG
*Hmgcr*	CACCTCTCCGTGGGTTAAAA	GAAGAAGTAGGCCCCCAATC
*Pparg*	GCCCTTTGGTGACTTTATGGA	GCAGCAGGTTGTCTTGGATG
*Pck1*	AAAAGCCTTTGGTCAACAAC	AAACTTCATCCAGGCAATGT
*Scd1*	CTGTACGGGATCATACTGGTTC	GCCGTGCCTTGTAAGTTCTG
*Srebf1*	ATCCAGGTCAGCTTGTTTGCGATG	TGGACTACTAGTGTTGGCCTGCTT

### Protein Extraction and Western blotting

Cell or tissue proteins were extracted with cell lysis buffer (Cell Signaling Technology) and protein concentration was determined using the DC Protein Assay kit (Biorad, Hercules, CA, USA). Equal amounts of proteins (∼25 µg) were loaded onto 4–15% Criterion TGX gels (Bio-Rad, Hercules, CA), resolved by SDS elecrophoresis, and then transferred to nitrocellulose membranes for immunoblotting. Membranes were blocked with 5% skimmed milk in Tris-buffered saline containing 0.05% Tween 20 before incubation for overnight at 4°C with primary antibodies. Immunoreactive proteins were visualized by peroxidase-labeled secondary antibodies and ECL system (Western Lightning Chemiluminescence Reagent, Perkin-Elmer, MA, USA). Equal loading of proteins was confirmed by stripping and restaining the membranes with β-actin antibodies. Band intensities were quantified using a densitometer (Quantity One, Bio-Rad, Hercules, CA).

### Liver and Adipose Histology

Liver or white adipose tissues (WAT) from the epididymal fat pads were rapidly removed, fixed in 10% NBF overnight and embedded in paraffin. Tissue blocks were sectioned 5 µm thick and stained with hematoxylin-eosin (H&E). In addition, frozen portions of liver samples from each group were also embedded in OCT compound (Sakura Finetek, Torrance, CA, USA), sliced and stained with Oil Red O. Stained slides were observed and photographed under bright field microscope (AX70, Olympus, Tokyo, Japan) equipped with a digital camera (Retiga Exi, Qimaging, Surrey, BC, Canada). Quantification of the Oil Red O staining was done by histologic scoring (n = 8 animals per group) using the color segmentation function of ImagePro Plus 6.3 software (Media Cybernetics Inc., Bethesda, MD, USA). Similarly, the sizes of the epididymal WAT were estimated using ImagePro Plus. In brief, 200 random adipocytes from representative photomicrograph sections of each mouse (n = 8 mice per group) were analyzed for cell size using the software.

### Statistical Analyses

Statistical analyses were performed using Prism (GraphPad, San Diego, CA, USA). Body weight changes were compared between groups using repeated measure two-way ANOVA coupled with post hoc Tukey's test. All other parameters measured were analyzed by one-way ANOVA followed by Tukey's test for multiple comparisons and unpaired two-tailed t-tests for simple pairs. All data are stated as mean ± SEM. Differences between means are considered statistically significant when the *P*<0.05.

## Results

### SR4 reduces body weight and fat mass in HFD obese mice without affecting food intake

**Figure 1:**
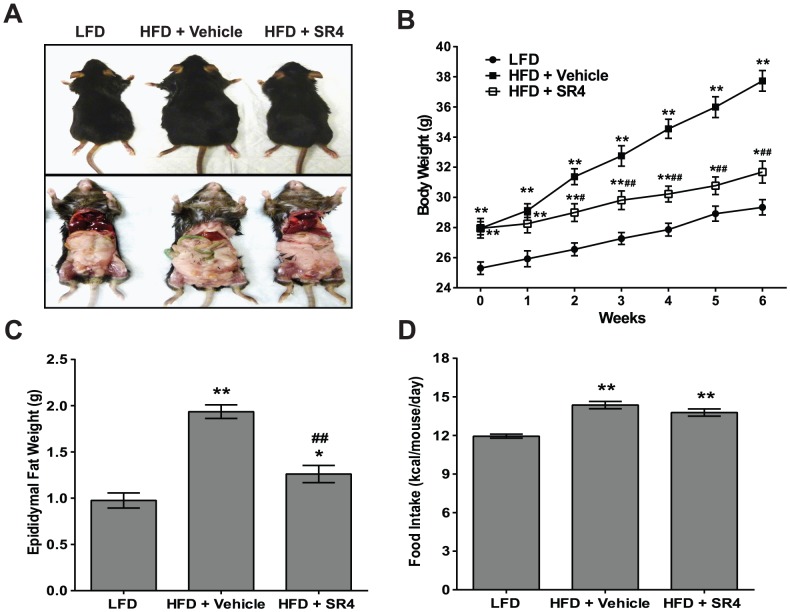
SR4 decreases body weight and fat mass in HFD obese mice. Representative mice in each treatment group depicting gross images of whole body shape (top) and abdominal fat (bottom) **(panel A**). Body weights **(panel B**), epididymal fat weight **(panel C**), and daily food intake per animal in each group (n = 8-12 animals per group) **(panel D**). Data represent mean ± SEM. *p<0.05 *vs.* LFD **p<0.01 *vs.* LFD; ^#^ *p*<0.05 *vs.* HFD vehicle; ^##^ *p*<0.01 *vs.* HFD vehicle.

Figure 1A shows representative mice fed either LFD or HFD treated with vehicle (corn oil) or SR4 (5 mg/kg b.w.) for 6 weeks orally. Compared with age-matched mice on LFD, HFD mice were significantly heavier throughout the duration of the study, with 22% more weight at week 6 (37.7±0.7 *vs*. 29.3±0.5 g, n = 13, *p*<0.01). Intermittent treatment with SR4 (3x/week) markedly reduced the body weights of HFD obese mice by 16% (*p*<0.01) compared with vehicle alone at the end of 6 weeks treatment (**Fig. 1B**). Significant weight reduction in HFD mice were observed as early as 2 weeks of treatment with SR4. Additionally, HFD mice also had ∼2-fold higher epididymal fat weight as compared to LFD mice (**Fig. 1C**). SR4-treated HFD mice had significant reduction (∼35%, *p*<0.01) in epididymal weight. Analysis of the food intake showed that SR4-treated HFD mice had similar amount of food consumption per day as that of vehicle-treated HFD mice, suggesting that the body weight and fat mass reduction in SR4-treated HFD mice were not associated with less caloric intake (**Fig. 1D**).

### SR4 improves glycemic control and dyslipidemia

**Figure 2:**
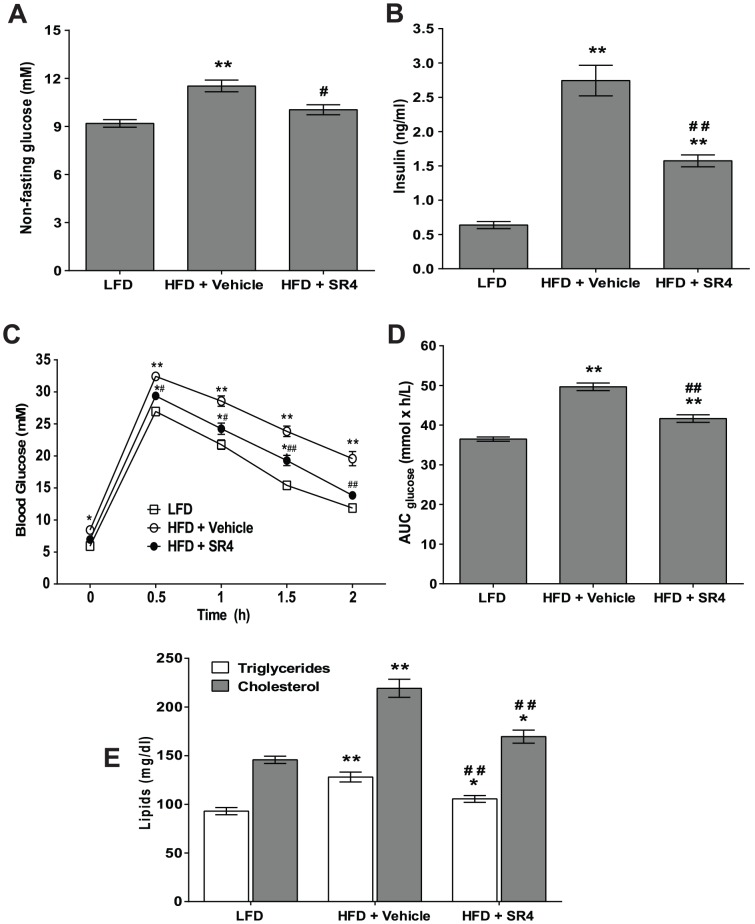
SR4 improves glycemic control and dyslipidemia in HFD obese mice. Plasma levels of non-fasting glucose **(panel A**) and insulin **(panel B**) were measured at the end of the 6-week treatment period. At week 5, mice were fasted overnight and GTT was performed **(panel C**) and glucose AUC integrated from 0–120 min for each mouse was calculated using GraphPad Prism software (n = 10 animals per group) **(panel D**). Plasma levels of cholesterol and triglycerides were also measured at the end of the experimental treatment **(panel E**). Data represent mean ± SEM. *p<0.05 *vs.* LFD; **p<0.01 *vs.* LFD; ^#^ *p*<0.05 *vs.* HFD vehicle; ^##^ *p*<0.01 *vs.* HFD vehicle.

Since obesity is one of the key risk factors for insulin resistance as well as T2D, the effects of SR4 on glucose and insulin sensitivity were tested. HFD mice exhibited mild hyperglycemia compared with age-matched LFD mice, with basal non-fasting glucose levels ranging from 9.2±0.2 mM ***vs*** *.* 11.5±0.4 mM respectively, at week 6. Administration of SR4 in HFD mice resulted in a significant reduction in plasma glucose to 10.0±0.3 mM (*p*<0.01; **Fig. 2A**). Levels of plasma insulin were also higher in HFD mice compared with LFD mice, and SR4 treatment reduced these insulin levels significantly (*p*<0.01, **Fig. 2B**). To further investigate insulin-resistance caused by HFD, we performed intraperitoneal glucose tolerance tests at week 5 of the experiment (**Fig. 2C**) after 16 h fast, and glucose tolerance was quantified as the area-under-curve (AUC) integrated from 0–120 min (**Fig. 2**D). As shown in these figures, HFD mice exhibited impaired glucose tolerance. SR4 treatment of these HFD mice significantly improved their glycemic control after the glucose challenge; glucose AUC was significantly suppressed by 16.7% compared with vehicle (*p*<0.01). Consistently, in T2D *db/db* mice, SR4 ameliorated glucose intolerance and insulin sensitivity (data not shown). Together, these results clearly indicate that SR4 could improve insulin sensitivity and glucose metabolism in obese and/or diabetic animals.

HFD mice also exhibited hyperlipidemia as evidenced by increased levels of circulating plasma triglycerides and cholesterol compared with LFD mice. SR4 treatment significantly lowered both circulating triglyceride (by 17.6%, *p*<0.05) and cholesterol (by 22.6%, *p*<0.01) in HFD obese mice (**Fig. 2E**). Collectively, these results show that SR4 affects both glucose and lipid metabolism in HFD obese mice.

### SR4 prevents adipose hypertrophy and normalizes adipokine levels

**Figure 3:**
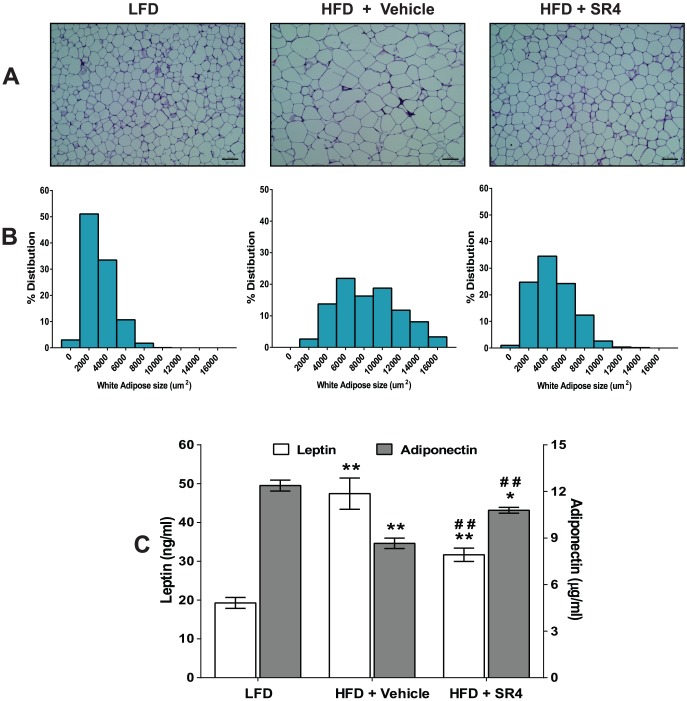
SR4 decreases adipose tissue hypertrophy and affects circulating adipokine levels in HFD obese mice. Representative photomicrograph images of sections of WAT (epididymal fat pad) stained with H&E from each treatment group (original magnification, 200×; scale bar, 100 um) **(panel A**). Cell size distribution of adipose cells from inguinal fat: The mean surface area and the frequency distribution were calculated based on at least 200 random cells from each mice (n = 8 animals per group) **(panel B**). Levels of plasma leptin and adiponectin at the end of experimental treatment (n = 8 animals per group) **(panel C**). Each bar represents mean ± SEM. *p<0.05 *vs.* LFD; **p<0.01 *vs.* LFD; ^#^ *p*<0.05 *vs.* HFD vehicle; ^##^ *p*<0.01 *vs.* HFD vehicle.

Histological analysis of WAT revealed that HFD mice had larger white (epididymal) adipocytes relative to LFD mice (**Fig. 3A**). Morphometric analyses of adipocyte distribution along with their sizes showed that HFD mice had higher population of bigger adipocytes in WAT than LFD mice (8884±136 um^2^ *vs.* 3108±39 um^2^) (**Fig. 3B**). SR4 treatment of HFD mice resulted to marked decrease in the overall size of the adipocytes (4228±54 um^2^) in the epididymal WAT; SR4 increased the population of smaller sized adipocytes and decreased the population of the larger-sized adipocytes.

HFD-induced obesity is also known to be strongly associated with the levels of adipokines such as leptin and adiponectin, both of which are secreted from WAT [29]. As shown in Figure 3C, circulating plasma levels of leptin in HFD mice was ∼2-fold higher than that of LFD mice (47.4±4.0 *vs.* 19.3±1.4 ng/ml, *p*<0.01). In contrast, the HFD plasma adiponectin concentration was ∼30% lower than that of LFD mice (8.6±0.4 *vs*. 12.4±0.4 ng/ml, *p*<0.01). SR4 treatment significantly decreased circulating leptin while increasing the adiponectin levels in HFD mice (*p*<0.01, **Fig. 3C**).

### SR4 reverses hepatic steatosis, decreases liver triglycerides and normalizes liver enzymes

**Figure 4:**
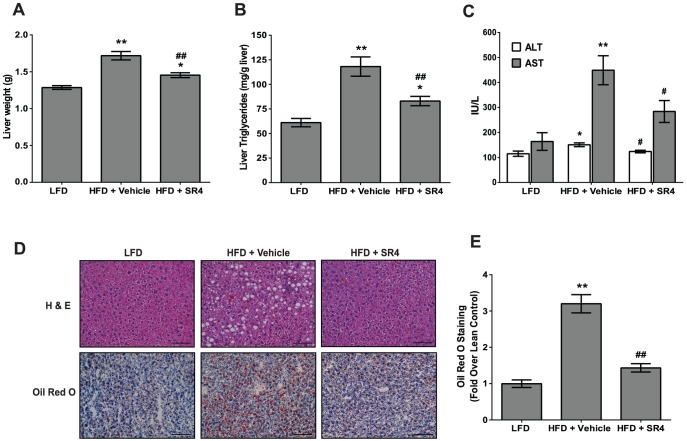
SR4 prevents hepatic lipid accumulation and fatty liver in HFD obese mice. Liver weights **(panel A**), total liver triglyceride contents **(panel B**), and plasma concentrations of liver enzymes ALT and AST **(panel C**) at the end of the treatment period (n = 8 mice per group). Representative photomicrographs of H&E staining of liver sections from each treatment group, original magnification, 100×; scale bar, 100 um **(upper panel D**). Representative photomicrographs of Oil Red O staining of liver sections from mice in each treatment group **(lower panel D**) and Oil red O staining quantification was done with Image Pro Plus 6.3 software using 3-4 random microscope field sections per animal sample and presented as fold over control (n = 8 animals per group) **(panel E**). Results represent mean ± SEM. *p<0.05 *vs.* LFD; **p<0.01 *vs.* LFD; ^#^ *p*<0.05 *vs.* HFD vehicle; ^##^ *p*<0.01 *vs.* HFD vehicle.

Since obesity is often accompanied by the development of fatty liver, we next examined the liver pathology of LFD and HFD mice upon SR4 treatment. Compared with age-matched LFD mice, HFD mice had significant increase in net liver weight by ∼38% (*p*<0.01, **Fig. 4A**). Biochemical analyses also revealed that HFD had marked increased in hepatic triglyceride contents, accompanied by elevated plasma levels of liver enzymes such as ALT and AST (**Figs. 4B and C**). Treatment with SR4 led to significant reductions in net liver weight (*p*<0.01), in liver lipid content (*p*<0.01), as well as the plasma levels of the two liver enzymes in HFD mice (*p*<0.04). To investigate further the effects of SR4 on hepatic steatosis, we examined the histology of the liver of SR4-treated mice. H&E stainings of liver sections revealed that HFD mice, in comparison with LFD mice, had numerous lipid-filled vacuoles and “foamy” cells, which are characteristics of extensive hepatic steatosis that develop in these mice (**Fig. 4D**, upper panel). Morever, Oil Red O stainings strongly showed accumulation of numerous larger fat droplets in these HFD mice (**Fig. 4D**, lower panel). In contrast, hepatocellular vacuolation was not observed from SR4-treated HFD mice and only scattered smaller Oil-Red O stained fat droplets were detected in the liver of these animals, almost similar as in LFD control mice (**Fig. 4D**). Quantitative scoring of Oil Red O stained specimens showed SR4 treatment significantly decreased liver droplet contents in HFD mice compared with vehicle treatment (*p*<0.01, **Fig. 4E**). The results indicate that SR4 improves the overall pathology of the liver and significantly prevented HFD-induced hepatic steatosis.

### SR4 inhibits hepatic lipogenic and gluconeogenic pathways

**Figure 5:**
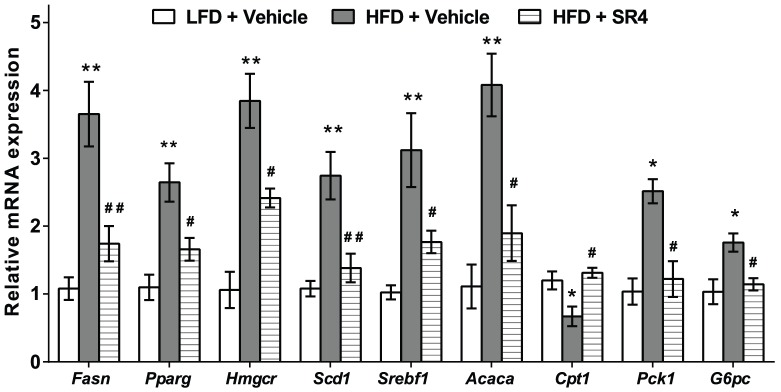
SR4 regulates expression of genes involved in lipid and glucose metabolism. Total RNA was isolated from liver of LFD lean or HFD obese mice treated with vehicle or SR4. Relative mRNA levels of liver lipogenic and gluconeogenic genes were determined using real time RT-PCR and quantified using the comparative Ct method with β-actin as internal control. Two independent RT-PCR experiments were performed (n = 4 animals per group). Each bar represents mean ± SEM, *p<0.05 *vs*. LFD; **p<0.01 *vs.* LFD; ^#^ p<0.05 *vs*. HFD vehicle; ^##^ *p*<0.01 *vs*. HFD vehicle.

To determine the molecular mechanisms involved in the inhibitory effect of SR4 on HFD-induced hepatic steatosis, we measured the expression of several genes involved in hepatic lipid and glucose metabolism in LFD and HFD mice upon SR4 treatment. Compared with the LFD mice, HFD mice had significant increase in the expression of most genes involved in fatty acid and cholesterol synthesis, including acetyl-Coenzyme A carboxylase (*Acaca*), fatty acid synthase (*Fasn*), peroxisome proliferator-activated receptor gamma (*Pparg*), sterol regulatory element binding protein-1c (*Srebf1*), carnitine palmitoyltransferase 1a (*Cpt1a*), stearoyl-Coenzyme A desaturase 1 (*Scd1*), and 3-hydroxy-3-methyl-glutaryl-CoA reductase (*Hmgcr*). In parallel with reduced fat content, SR4 treatment of HFD obese mice led to decreased expression of these lipogenic genes to towards normal control levels (**Fig. 5**). Moreover, SR4 also showed a tendency to suppress expression of gluconeogenic genes phosphoenolpyruvate carboxykinase 1 (*Pck1*) and glucose-6-phosphatase (*G6pc*) in liver of HFD mice. These data indicate that SR4 might alleviate HFD-induced metabolic abnormalities, at least in part, through regulating the expression of a certain set of genes associated with hepatic lipid and glucose metabolism.

### SR4 stimulates AMPK phosphorylation in liver and adipose tissues

**Figure 6:**
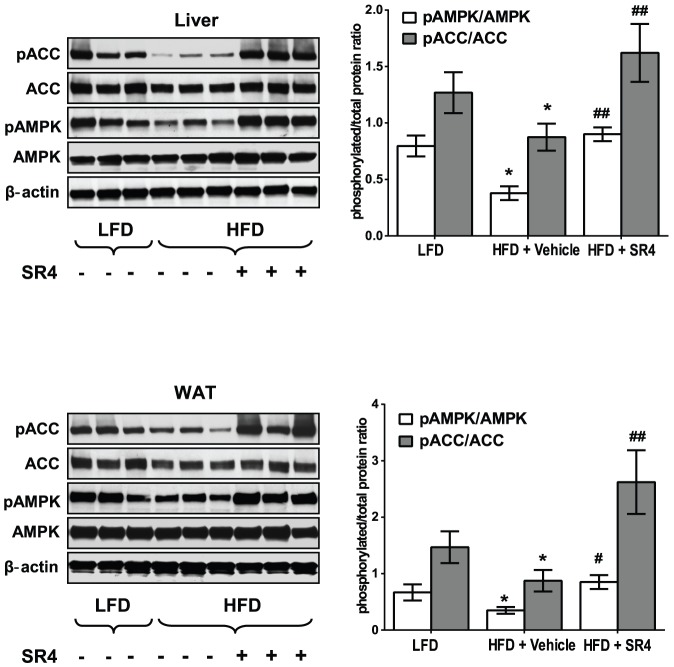
SR4 activates AMPK in liver and WAT. Representative Western blots analyses of protein lysates from liver and epididymal WAT. Total protein lysates (n = 3 animals in each group) were subjected to SDS-PAGE and immuno-blotted with antibodies specific for phosphorylated and total AMPK, phosphorylated and total ACC or β-actin. The pAMPK/AMPK and pACC/ACC ratios were quantified using a densitometer. At least two independent experiments were performed. Each bar represents mean ± SEM, *p<0.05 *vs*. LFD; ^#^ *p*<0.05 *vs.* HFD vehicle; ^##^ *p*<0.01 *vs.* HFD vehicle.

AMPK activation is known to modulate many aspects of hepatic glucose and lipid metabolism, as well as lipogenic pathways in WAT [10], [11]. As shown in Figure 6, chronic treatment with SR4 induced the phosphorylation of AMPK in the liver and WAT of HFD mice, concomitant with increased phosphorylation of its target protein ACC. These data indicate that SR4 is a potent activator of AMPK *in vivo*, which would result in its beneficial effects on glucose and lipid metabolism.

### SR4 indirectly activates AMPK independent of calcium signaling and LKB1 *in vitro*

**Figure 7:**
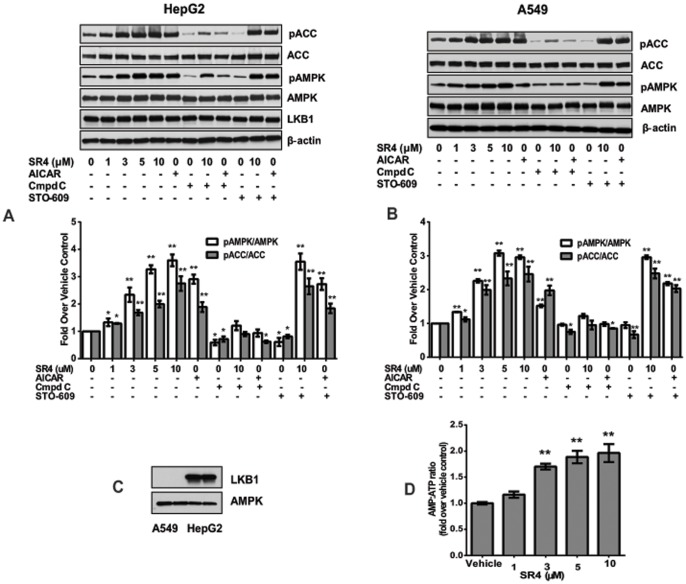
SR4 activates AMPK independent of upstream kinases LKB1 and CaMKKβ. Representative Western blots analyses showing SR4 dose-dependently activates AMPK in human liver hepatocellular carcinoma (HepG2) **(panel A**) and human lung adenocarcinoma (A549) cells **(panel B**). Cells were treated with SR4 (0, 1, 3 5, 10 µM) or the AMPK activator AICAR (2 mM) in the presence or absence of the AMPK inhibitor Compound C or CaMKKβ inhibitor STO-609 (added to the cells 30 min prior to the addition of SR4 or AICAR). After 4 h, cells were collected, lysed, and total cell lysates subjected to Western blot analyses. Densitometric quantitation was performed on each blot and the fold changes were compared with vehicle (DMSO) control. At least three independent experiments were performed, *p<0.05 *vs.* vehicle control; **p<0.01 *vs*. vehicle control. Western blots showing the lack of LKB1 protein in A549 cells **(panel C**). Intracellular AMP: ATP ratios in HepG2 cells following 4 h treatment with various concentrations of SR4 **(panel D**). Results are shown as mean ± SEM of three independent experiments with three replicates each *p<0.05 *vs.* DMSO vehicle; **p<0.01 *vs.* DMSO vehicle.

We further examined the molecular mechanisms by which SR4 activates the AMPK pathway. Consistent with COH-SR4-induced activation observed in mouse liver and WAT, liver HepG2 cells treated with SR4 showed a concentration-dependent increase in phosphorylation of AMPK and its target protein ACC. The effects of SR4 were comparable, if not better than the activating effect of AICAR (2 mM), an analog of AMP and a well-known cell permeable activator of AMPK (**Fig. 7A**). To further characterize the mechanisms leading to AMPK activation, HepG2 cells were pre-treated with the AMPK inhibitor Compound C, or with STO-609, a specific inhibitor of the Ca^2+/^calmodulin-dependent protein kinase kinase (CaMKKβ). Compound C treatment diminished the phosphorylation of AMPK and ACC by both COH-SR4 and AICAR. In contrast, STO-609 did not significantly reduce AMPK and ACC phosphorylation in SR4 or AICAR-treated cells, indicating that AMPK activation of both of these compounds is independent on intracellular Ca^2+^ signaling. In addition, we also pre-treated A549 cells, an LKB1-deficient cell line (**Fig. 7C**) with Compound C and ST0-609 before incubation with either COH-SR4 or AICAR. Interestingly, we observed very similar effects of SR4 and AICAR on the phosphorylation of both AMPK and ACC in this cell line compared with HepG2 (**Fig. 7B**), suggesting that SR4 activates AMPK independent of both LKB1 and CaMKKβ.

Since AMPK activation by SR4 is independent of both upstream kinases, we determined next whether SR4-dependent AMPK activation is associated with changes in intracellular nucleotide levels, specifically the AMP: ATP ratio. HepG2 cells were treated with vehicle or SR4 for 1 h and the concentrations of nucleotides were measured by HPLC. Treatment with SR4 increased the AMP: ATP ratio in a concentration-dependent fashion (**Fig. 7D**), similarly to what we observed earlier on 3T3-L1 adipocytes [27]. These data further confirms that SR4 indirectly activates AMPK by increasing the cellular AMP: ATP ratio.

## Discussion

Our current data describes the pharmacological activity of a novel small molecule dichlorophenyl urea compound, SR4, in HFD obese mice. Consistent with previous studies, compared with LFD lean mice, HFD mice have significantly increased body weight and fat mass, leading to moderate hyperglycemia, glucose intolerance and hyperinsulinemia. In addition, these mice have elevated plasma lipids, hepatic lipid triglycerides and promoted the development of fatty liver [30], [31]. Treatment of these mice with SR4 ameliorated the gain in body weight and fat mass without affecting food intake, improved glucose control and insulin sensitivity, and decreased circulating plasma lipids. SR4 also prevented hepatic steatosis in HFD mice, concomitant with decreased liver lipid triglycerides, increased leptin and decreased adiponectin levels, and normalization of liver enzymes ALT and AST. Based on our data, these overall improvements in glucose and lipid homeostasis were more likely due to the ability of SR4 to activate AMPK in HFD obese mice.

The liver is a key organ in lipid and glucose metabolism, where it is the major site for storage and release of carbohydrates as well as synthesis of fatty acids. Derangement in lipid and glucose metabolism in the liver, characterized by enhanced production and impaired catabolism often caused by elevated levels of hepatic enzymes synthesizing lipid and glucose, contributes to obesity and insulin resistance, leading to diabetes, liver disease and metabolic syndrome [32]–[34]. While AMPK-mediated effects are observed in multiple tissues, accumulating evidence has demonstrated significant role of AMPK in balancing lipid and glucose production *via* regulation of activity of enzymes associated with lipid and glucose metabolism in maintaining whole-body energy homeostasis [10], [11]. AMPK directly phosphorylates and inactivates a number of metabolic enzymes involved in hepatic lipid metabolism, including ACC and HMGR. Phosphorylation of ACC1 at Ser^79^ and ACC2 at Ser^218^ by AMPK leads to inhibition of ACC activity and decreased malonyl-CoA content, leading to reduced fatty acid biosynthesis and increased CPT1, the rate-limiting step in the import and oxidation of fatty acids in mitochondria [35]. AMPK activation also leads to phosphorylation and inhibition of HMGCR, which blocks the conversion of HMG-CoA to mevalonate thereby reducing cholesterol levels [9]. Additionally, AMPK phosphorylation and activation also reduces the expression of the transcription factor SREBP-1c which modulates the expression of genes involved in the fatty acid synthetic pathway, such as FAS, SCD1, glycerol-3-phosphate acyltransferase (GPAT), and ACC1 and ACC2 [10], [17]. Recently, AMPK was shown as a direct upstream kinase that binds to and phosphorylates SREBP-1c and -2, inhibits their cleavage, nuclear translocation, and transcriptional activity, and ultimately suppresses lipogenesis and lipid accumulation in hepatocytes [12]. Over-expression of the lipogenic genes such as SREBP-1c, FAS, and ACC1 in obese individuals have been shown to be strongly associated in the development of chronic NAFLD, a broad spectrum of liver abnormalities ranging from simple hepatic steatosis to a more severe form, non-alcoholic steatohepatitis (NASH), which is associated with hepatocyte damage, chronic inflammation, and fibrosis, and may progress to cirrhosis and liver failure [36]. In the present study, we observed SR4 activates AMPK in the liver of HFD obese mice and reduced the mRNA expression of key lipogenic genes including *Acaca*, *Fasn*, *Scd1*, *Cpt1a*, *Pparγ*, *Hmgcr*, as well as the transcription factor *Srebf1*, leading to the suppression of *de novo* lipid production and eventually inhibition of hepatic steatosis. These results are consistent with other AMPK activators such AICAR [37], berberine [23], resveratrol [38] and glabridin [24], which showed similar anti-obesity effects, associated with decreased expression of hepatic lipogenic enzymes and reduced lipid synthesis *in vivo*. Thus, it is likely that SR4-stimulated AMPK activation alters the expression of genes involved in lipid metabolism, which would ameliorate lipid dysfunction, as well as influence glucose regulation in obese animals.

For developing potential treatment to combat obesity, it is desirable that body weight loss is accompanied by a reduction in fat mass. Increase in adipose tissue mass may be due to an increase in the number of adipose cells, or an increase in adipose size due to fat storage. In the present study, aside from preventing dyslipidemia, SR4 administration leads to a reduction on epidydimal fat weight and adipose hypertrophy, the latter playing a central role in the formation of adipose mass tissues. Previously, we reported that SR4 inhibited adipogenesis in mouse 3T3-L1 cells by modulating adipogenic and lipogenic genes *via* the AMPK-mTORC1 pathway [27]. Here, AMPK activation was also observed in epidydimal WAT, but no change in gene expression of adipogenic transcription factors *Pparg* and *Srebf1* was detected in these adipose tissues (data not shown), indicating the reduction in epidydimal weight by SR4 is not due to blockage of adipocyte differentiation as observed *in vitro* in 3T3-L1 cells, but more likely associated with lipid accumulation and increase in size of adipose cells (hypertrophy) as seen in our histological stainings. Nonetheless, to what extent SR4 affects the mTOR pathway in these obese mice remains to be investigated, but targeting mTOR pathway may have some beneficial effects on obesity. Indeed, mice with adipose-specific loss of the mTORC1 are lean and resistant to HFD-induced obesity [39], while chronic pharmacological inhibition of mTOR by its specific inhibitor rapamycin exhibited anti-obesity effects in HFD mice [40].

SR4 treatment also improved glycemic control and glucose tolerance in HFD obese mice (as well in type 2 diabetic *db/db* mice, data not shown) as evidenced by significant improvement in non-fasting glucose levels, glucose tolerance tests, and decrease in circulating plasma insulin levels. Although reduced adiposity and normalization of adipokine levels are also likely to contribute to the preservation of insulin sensitivity and better glucose regulation observed on SR4-treated HFD obese mice, these beneficial effects could also be associated with the ability of the compound to activate AMPK and target hepatic gluconeogenesis. Indeed, previous experiments have shown the physiological importance of hepatic AMPK for glucose homeostasis; liver-specific deletion of AMPKα2 caused hyperglycemia, glucose intolerance, reduced muscle glycogen synthesis, and chronically elevated free fatty acid levels as a result of enhanced gluconeogenesis [41], [42], while short-term over-expression of a constitutively active form of AMPK in the liver results in reduced blood glucose and increased hepatic fatty acid oxidation, suggesting a preference for fatty acid utilization in supplying energy needs [43]. In these studies, the gene expression of liver gluconeogenic enzymes such as *P6k1* and *G6pc* were significantly affected, similar to what we observed in our present study. Previous reports also showed that pharmacological activation of AMPK leads to the phosphorylation of carbohydrate response element–binding protein (ChREBP) at Ser^568^, which caused a decrease in its DNA binding activity and subsequent transcriptional inhibition of glucose responsive genes [13]. Similarly, a new transcription factor phosphorylated by AMPK, AICAR response element binding protein (AREBP), was identified which binds to the promoter element of PEPCK and represses its gene expression [44]. Whether SR4 affects these two transcription factors *via* AMPK activation to suppress hepatic gluconeogenesis needs further analysis.

It is well documented that AMPK activation is mediated by several different mechanisms such as changes in AMP/ATP ratio, intracellular Ca^2+^ and activities of AMPK upstream kinases [45]. Many of the well-known pharmacological drugs (biguanides, thiazolinediones, statins) as well as phytochemicals (berberine, resveratrol, glabridin, genestein) with varying structures activate AMPK indirectly by influencing mitochondrial activity [7], [10], [11], [16]. Our present studies indicate SR4 would activate AMPK independent of CAMKKβ- or LKB-activation. In addition, we observed that SR4 increased the AMP/ATP ratio in HepG2 cells, consistent with our observations earlier with 3T3-L1 adipocytes [27]. Thus, these data imply that increase in intracellular AMP/ATP ratio by SR4 would be the major molecular mechanism responsible for AMPK activation. We are currently investigating the effects of SR4 in the mitochondria, specifically its effects on mitochondrial respiration and associated mitochondrial complexes.

As mentioned earlier, SR4 was originally identified as a novel anti-cancer agent that showed strong anti-proliferative activites in many types of human cancers *in vitro*, as well as in animal xenograft experiments [25], [26], [46]. Recently, we found that SR4 dose-dependently activates AMPK in various types of human and animal cancer cell lines (leukemia, lung, pancreatic, liver, ovarian, melanoma, glioma, breast) (unpublished data), as well as 3T3-LI adipocytes [27]. Knockdown of AMPK *via* siRNA interference diminished the effects of SR4 on these cells. Also, as demonstrated in the present study, AMPK activation was observed in the liver and WAT of SR4 treated HFD obese mice, consistent with increased AMPK activation we found in resected tumor tissues and Western blot analyses of tumor tissue lysates in melanoma [26] and lung cancer xenografts [46]. Taken together, these results clearly indicate that the effects of SR4 are mediated *via* AMPK activation. Several other AMPK activators including berberine, metformin and resveratrol that are known to have anti-diabetic/anti-obesity effects also have recently demonstrated potent anti-cancer effects [16]–[18], [47]–[49]. Thus, our present data adds to the growing number of evidence demonstrating that AMPK activation is indeed a viable target for the treatment of metabolic diseases as well as cancer.

As with most drug targets, treatment with AMPK activators (whether for metabolic disorders or for cancer), might cause some undesirable side effects. Some of the reported detrimental effects of AMPK activation included a possible tumor-promoting effect thought to be dependent upon the stage in the development of the tumor [50], as well as increased food intake and body weight gain *via* activation of the hypothalamic AMPK in rodents [51]. In our syngeneic as well as xenograft melanoma and lung cancer studies, oral feeding of SR4 showed no changes in blood chemistries [26], [46]. In addition, SR4-treated tumor-bearing animals survived longer and had no significant effects on body weigh and food intake. Similarly, C57BL/6J mice on a low fat diet treated with SR4 showed no marked increase in food consumption and body weight gain (data not shown). Taken together, these observations indicate that SR4 is well-tolerated with no overt toxicity in rodent models.

In conclusion, SR4 ameliorates adiposity and metabolic disorders in HFD obese animals. These effects of SR4 are likely associated with its ability to stimulate AMPK and influence both lipid and glucose metabolism. Further studies are required to expand our knowledge on this class of novel AMPK activator as potential therapeutic agent for obesity and related metabolic diseases.
